# A blind image super-resolution network guided by kernel estimation and structural prior knowledge

**DOI:** 10.1038/s41598-024-60157-9

**Published:** 2024-04-25

**Authors:** Jiajun Zhang, Yuanbo Zhou, Jiang Bi, Yuyang Xue, Wei Deng, Wenlin He, Tao Zhao, Kai Sun, Tong Tong, Qinquan Gao, Qing Zhang

**Affiliations:** 1 The College of Physics and Information Engineering, Fuzhou University, Fuzhou, 350108 China; 2 The Beijing Radio and TV Station, Beijing, 100022 China; 3 University of Edinburgh, Edinburgh, UK; 4 The Imperial Vision Technology, Fuzhou, 350000 China; 5 The College of Computer Engineering, Jimei University, Xiamen, 361021 China

**Keywords:** Electrical and electronic engineering, Software

## Abstract

The goal of blind image super-resolution (BISR) is to recover the corresponding high-resolution image from a given low-resolution image with unknown degradation. Prior related research has primarily focused effectively on utilizing the kernel as prior knowledge to recover the high-frequency components of image. However, they overlooked the function of structural prior information within the same image, which resulted in unsatisfactory recovery performance for textures with strong self-similarity. To address this issue, we propose a two stage blind super-resolution network that is based on kernel estimation strategy and is capable of integrating structural texture as prior knowledge. In the first stage, we utilize a dynamic kernel estimator to achieve degradation presentation embedding. Then, we propose a triple path attention groups consists of triple path attention blocks and a global feature fusion block to extract structural prior information to assist the recovery of details within images. The quantitative and qualitative results on standard benchmarks with various degradation settings, including Gaussian8 and DIV2KRK, validate that our proposed method outperforms the state-of-the-art methods in terms of fidelity and recovery of clear details. The relevant code is made available on this link as open source.

## Introduction

The task of image super-resolution (SR) is to reconstruct clear high-resolution images from low-resolution images. Image degradation is often considered as the inverse problem of SR, as it involves mathematically modeling the processes that deteriorate the quality of image. According to previous works^1–5^, the pipeline of degradation is typically modeled as Eq. (1).

1 $$\begin{aligned} y = (x *k_{h})_{\downarrow _{s}}+n, \end{aligned}$$

where *x* represents the high resolution (HR) image, while *y* corresponds to the low resolution (LR) image. The operator $*$ denotes the two-dimensional convolution operation and $k_{h}$ is the Gaussian kernel, $\downarrow _{s}$ means downsampling operation with a scale factor of *s*, *n* refers to additive Gaussian white noise (AGWN). The classical SR methods^6–8^ assumes that the degradation pipeline is a single bicubic downsampling. However, if the predefined degradation does not exactly match the practical situation, the reconstructed HR image may exhibit unpleasant artifacts^1^. Therefore, recovering shape edges and rich details in the case of LR images with unknown degradation^1,2,5,9–12^, is an extremely meaningful and challenging task.

The most common blind SR schemes are typically divided into two stages: the first stage is to model the kernel explicitly or implicitly through optimizing a deep neural network from the degraded image^1–5,9^, and the second stage inputs the LR image combined with additional degradation prior through the SR network to obtain reconstructed HR image. In first stage, the mismatch between estimated blur kernel and the actual one can lead to over-smoothed or over-sharpened results^1–3^. An available solution is to perform accurate estimation of the kernel^1,9^ and robust integration with the SR backbone^2,3,5^.

**Figure 1:**
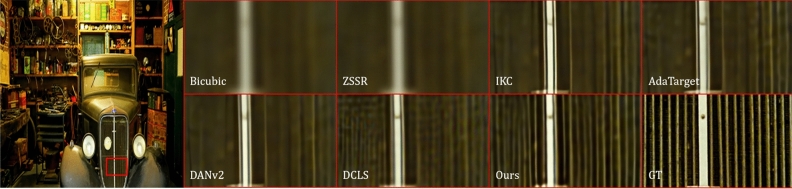
Blind super-resolution of Img100 from DIV2KRK^9^, for scale factor 4. Based on the fusion of local and global features, our method is effective in restoring sharp and clean edges, and outperforms previous state-of-the-art approaches such as ZSSR^13^, IKC^1^, AdaTarget^14^, DANv2^2^, and DCLS^3^.

Recent research^1–5,9^ has mainly concentrated on the first stage of kernel modeling. DCLS^3^ proposes a robust dynamic kernel estimation network and introduces a module to achieve degradation representation embedding. However, its SR network has limited ability to represent spatial features, making it difficult to recover structural information well. Fig. 1 shows the reconstruction results of state-of-the-art methods and our method for structural textures. It can be observed obviously that current methods lacks the combination of structural prior knowledge, making the ambiguous details and edges in the recovered SR image.

It is broadly recognized that non-local operations^15,16^, which introduce self-similarity priors, are significant for recovering recurring textures within the same image. Moreover , the spatial attention and channel attention mechanisms can effectively capture local features. Motivated by these observations, we propose a network combined kernel estimation and structural prior knowledge that can leverage both local spatial and global features to boost reconstruction performance for images with high self-similarity. To be specific, we employ the deep constrained least squares^3^ (DCLS) block as the module to deblur the original feature $f_o$, in order to obtain a clean feature $f_c$. Next, we divide the original feature $f_o$ into two vectors along the channel dimension: $\widehat{f_o}$, and $\overline{f_o}$. These three vectors $f_c$, $\overline{f_o}$, and $\widehat{f_o}$, are together fed into a series of triple path attention blocks (TPAB) to perform deep feature extraction and utilize local spatial information to compensate for the gap caused by kernel estimation. Furthermore, the global texture fusion block (GTFB) adaptively adjusts the self-similarity scores of non-local features to achieve the embedding of global structural prior. We have performed several standard experiments on benchmarks with various degradation settings to evaluate our proposed method. The quantitative and qualitative results demonstrate that our network has excellent performance in all datasets, particularly for images with rich structural information. The main contributions of this paper are summarized as follows:

- We propose a blind SR network, capable of combining kernel estimation with structural prior knowledge to reconstruct the textures with high self-similarity.
- We employ a channel split strategy to take advantage of the original local spatial and channel features in order to compensate for artifacts generated by the kernel estimation and the deblurring operation.
- We design a global texture fusion block that aggregates local spatial features with non-local operations to enhance recovery performance in images with high self-similarity.
- Extensive experiments with various degradation settings demonstrate that our method achieves outstanding performance in the task of blind SR.

## Related work

### SR of bicubic and multiple degradation

The pioneering work of SRCNN^6^ has successfully motivated interest among researchers in the field of SR. Inspired by hierarchical architecture^7,8,17^ and robust loss function^11,12,18–21^, CNN-based methods have achieved outstanding performance on predefined bicubic downsampling in the SR task, while the degradation process in the real-world are generally unknown and complicated^11,12^. In practical applications, if the bicubic kernel assumed by classical methods does not match the actual degradation kernel, it will lead to unpleasant artifacts in the reconstructed SR image, severely affecting the visual perception quality. This discrepancy between the assumed kernel and the actual kernel give rise to domain gap^22–24^, which is a challenge in practical applications of SR.

Another approach to non-blind SR method^4,25–28^ is designed to super-resolve multiple types of degraded images with corresponding kernels. These methods make classical SR networks more robust and applicable to a wider range of real-world scenarios. FFDNet^25^ utilizes a noise level map as additional input, allowing it to handle various noisy images affected by different types of degradation. Similarly, SRMD^4^ proposes a kernel stretching strategy that incorporates the two degradation parameters, the blur kernel *k* and the noise level *n*, together with the LR as input to SR network. Zhang et al.^29^ combines learning-based methods with model-based methods to design an end-to end unfolding networks that can handle various types of degraded images with different scales. UDVD^27^ introduces dynamic convolution in the kernel estimation network, where the parameters of the filters can be dynamically adjusted based on the adaptivity of the input degraded image. KMSR^26^ utilizes generative adversarial networks to learn the distribution of kernels in real degraded images. Inspired by KMSR^26^, Son et al.^28^ propose an adaptive downsampling model that employs an unsupervised approach to simulate the actual degradation process of real-world images. They then synthesize paired data and develop an SR network capable of handling various types of degradation.

### SR of unknown kernel

The most common approach for the blind SR task is based on kernel estimation methods^1–5,9,30^. KernelGAN^9^ utilizes cross-scale image similarity to accomplish kernel estimation on specific images and combined it with a classical method^13^ to achieve blind reconstruction. MANet^30^ further investigates spatially variant blur kernels in order to super-resolve objection motion and out-of-focus in real world scenarios. Gu et al.^1^ use an iterative correction method to alleviate the effects caused by the mismatch between estimated result and practical kernel. Luo et al.^2,5^ adopt an end-to-end network to alternately optimize estimator and restorer. These two methods^1,2^ are effective but time-consuming owing to the elaborate optimization steps. DCLS^3^ reformulates a practical degradation model and proposes a deep constrained least squares module to operate deconvolution in order to achieve robust degradation awareness. In the aforementioned methods^1–3,5,9,22,23^, the solution is concentrated on modeling degradation either implicitly^22,23,31^ or explicitly^1–5,9,10,32^ without delving into the function of structural textures as prior knowledge. This may be a potential factor leading to the upper bound of blind SR performance.

**Figure 2:**
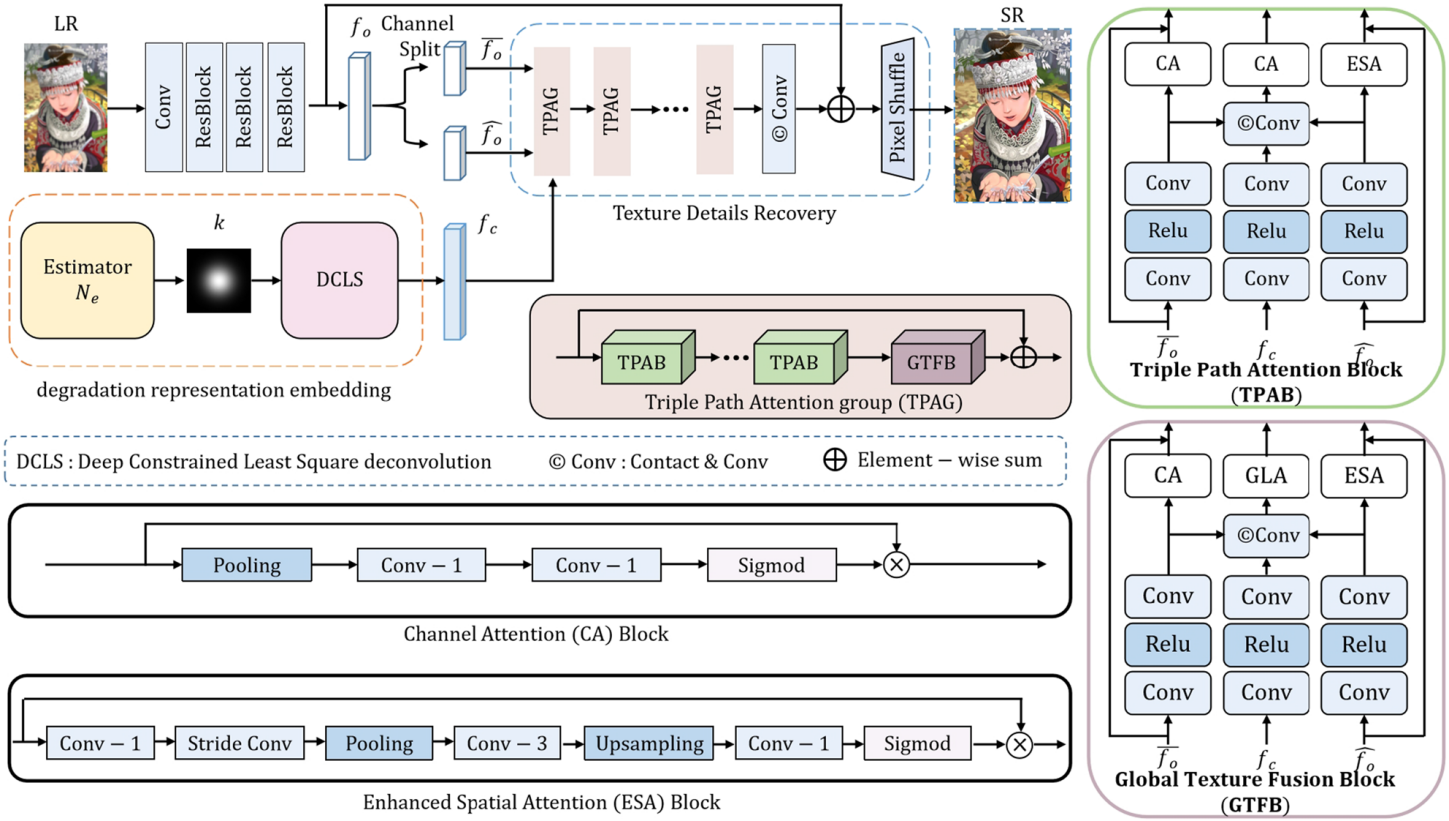
The overall architecture of our network and the structure of related blocks. Given an LR image, we first estimate the kernel *k*, and feed into DCLS module to achieve degradation presentation embedding. The triple path attention groups utilize the clean feature $f_c$ and the chunked original feature $\overline{f_o}$ and $\widehat{f_o}$ as input to restore the clean SR image.

## Method

### Architecture

In this subsection, we will introduce the overall architecture of our model. As shown in Fig. 2, our method mainly contains two stages: degradation representation embedding, and texture details recovery. The first stage includes the dynamic kernel estimation and deblurring operation based on the DCLS^3^ module. The estimator $N_e$ accomplishes robust kernel estimation from degraded LR image. Next, the LR image and the estimated blur kernel *k* are jointly input into the DCLS module for deblurring. Lastly, the clean and original shallow features are fed into the triple path attention network to achieve local and global features fusion, which consists of triple path attention blocks (TPAB) and global texture fusion blocks (GTFB). Details on the pipeline of our method and the relevant blocks will be described in the following subsections.

### Degradation representation embedding

**Figure 3:**
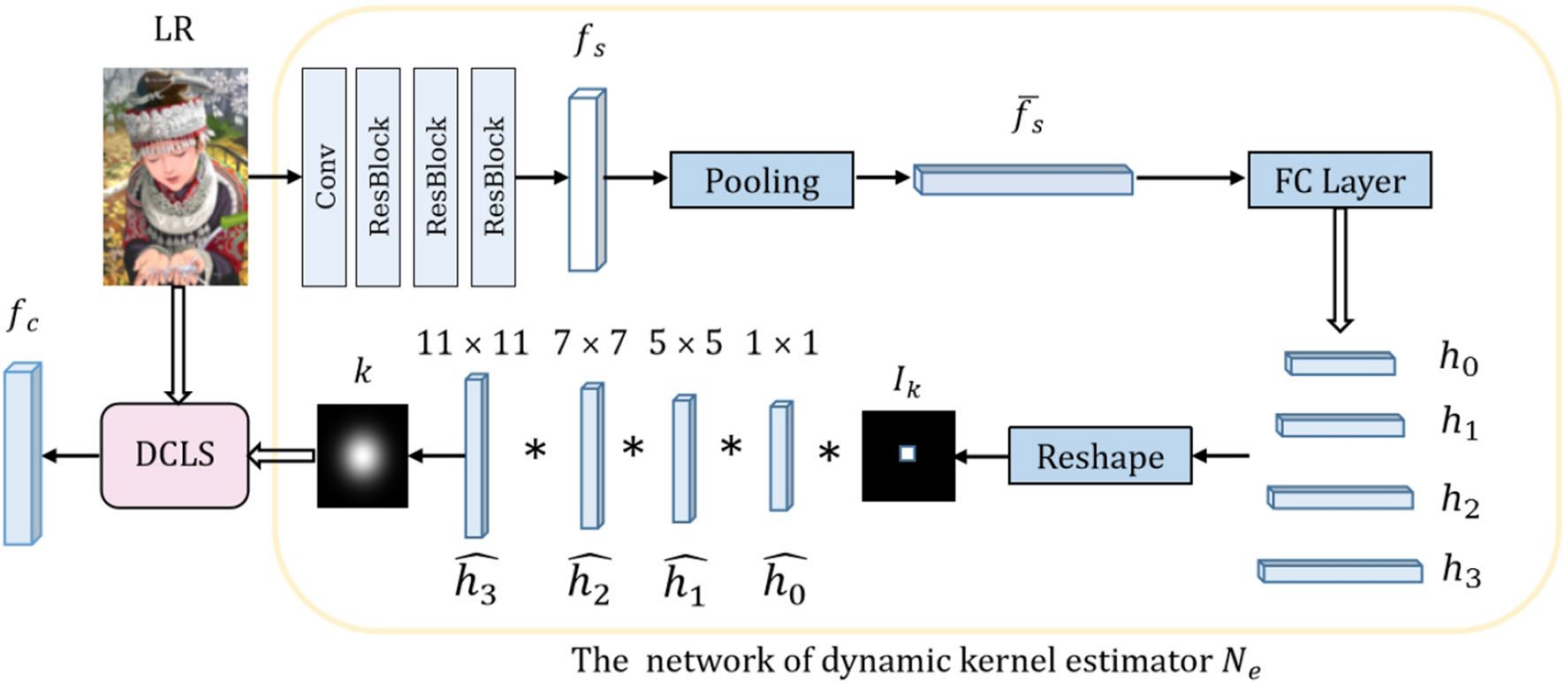
The overall architecture of dynamic kernel estimation. Given an LR image input, it first generate four specific filters. Then, these filters convolved sequentially with an identity kernel $I_k$ to produce a single kernel *k* with a larger receptive field corresponding kernel size.

Inspired by the work of^3^, our method employs the dynamic kernel estimation, as shown in Fig. 3. Given an LR image with unknown degradation as input, three residual blocks are applied to extract deep features $f_s$, followed by global average pooling to obtain the flattened features $\overline{f_s}$. The fully connected layer maps the specific degradation information to the four various filters, $\widehat{h_0}$ , $\widehat{h_1}$ , $\widehat{h_2}$ , and $\widehat{h_3}$ , with kernel sizes set to $11\times 11$, $7\times 7$, $5\times 5$ and $1\times 1$, respectively, to adjust the receptive filed consistency with the kernel sizes of predicted kernel *k*. The process of dynamic estimation is shown in Eq. (2).

2 $$\begin{aligned} k = I_k*\widehat{h_0}*\widehat{h_1}*\widehat{h_2}*\widehat{h_3}, \end{aligned}$$

where $I_k$ is the identity kernel, and $\widehat{h_0}$ , $\widehat{h_1}$ , $\widehat{h_2}$ , and $\widehat{h_3}$ are specific filters mapped from degradation information, *k* is the estimated kernel through Estimator $N_e$. The $I_k$ is sequentially convolved with these filters, enabling the parameters in network $N_e$ to vary with different degraded inputs. Meanwhile, the DCLS^3^ module utilizes deconvolutional operations to obtain clean feature as Eq. (3).

3 $$\begin{aligned} f_c = DCLS_{deconvolve}(f_{o},k), \end{aligned}$$

where $f_{o}$ represents the blurry original features extracted by a $3\times 3$ convolution layer and three residual blocks from the LR image, *k* is the kernel predicted by the network $N_e$, $f_{c}$ represents the deblurred clean features through the deconvolutional operation via the DCLS^3^ module.

### Texture details recovery

Even with introducing deconvolutional operation through the DCLS^3^ module, the damaged high-frequency information cannot be fully restored. Therefore, we propose a novel network that not only strongly extracts local features to compensate for the decline of high-frequency components but also incorporates non-local^15,16^ operation to fuse the local and global features.

Figure 2 illustrates the proposed SR network, mainly consists of the extraction process of original features and the fusion process of local features with global features. A $3\times 3$ convolutional kernel and three residual blocks without batch normalization^33^ is used to extract original features $f_o$ as Eq. (4).

4 $$\begin{aligned} f_o = h_{Reslobck}(h_{conv}(I_{LR})), \end{aligned}$$

where $I_{LR}\in {R^{H\times W\times C}}$ is an LR image as input, *H* and *W* represent the height and width of the patch that is cropped from a sub-image, and *C* is the RGB channels in the image.

In previous stages we have obtained clean features $f_c$. FAIG^34^ demonstrates that one branch network without degradation prior can achieve comparable performance to the two-branch method with degradation information. Although it may be reasonable to directly use the clean feature $f_c$ as input to the SR network for recovery, the offset of kernel estimation^9,30^ and insufficiency of deblurring function in the DCLS^3^ module would prevent the SR network from effectively restoring highly structured textures in the SR backbone. Therefore, we propose a Triple Path Attention Group (TPAG) to extract deep feature *f* as Eq. (6).

5 $$\begin{aligned} \psi (fc,\overline{f_o},\widehat{f_o})&= h_{GTFB}(h^n_{TPAB}(fc,\overline{f_o},\widehat{f_o})), \end{aligned}$$

6 $$\begin{aligned} f&=\psi _N(\psi _{N-1}(\psi _2(\cdots \psi _1((fc,\overline{f_o},\widehat{f_o})))), \end{aligned}$$

where the $\psi (fc,\overline{f_o},\widehat{f_o})$ represents TPAG that adopts the clean feature $f_c$, chunked original feature $\overline{f_o}$ and $\widehat{f_o}$ as additional inputs, $h_{GTFB}(h^n_{TPAB})$ means that the group is composed of *n* Triple Path Attention Blocks (TPAB) and one Global Texture Fusion Block (GTFB). *f* is the deep clean feature, *N* is the number of TPAG in our SR network.

In addition, we further refine the deep feature *f* through a $3\times 3$ convolutional layer with the original low-frequency feature $f_{o}$ connected through long skip connections^7,8,35,36^, as Eq. (7).

7 $$\begin{aligned} I_{SR} = h_{upsample}(h_{conv}(f)+f_{o}). \end{aligned}$$

Finally, pixel shuffle^37^ serves as the upsampling module and completes the mapping from feature maps to HR image $I_{SR}$.

### Triple path attention block

Deep SR networks contain specific filters that can handle various types and levels of degraded images^34^. These specific filters, which can be used to address corresponding degradation such as noise and blur, are located at different positions and branches within a single SR network. Channel attention^8,36,38,39^ and spatial attention^40,41^ mechanisms can enhance the local modeling ability. Therefore, we introduce these mechanisms as two branches in TPAB, allowing the network to strengthen its generalization and better handle different types of degradation.

The triple path attention blocks, consisting of residual channel attention and residual local spatial blocks, is shown in Fig. 2. The original shallow features $f_{o}$ are split into two feature maps $\overline{f_o}$ and $\widehat{f_o}$ along the channel dimension. They are combined with the deblurred clean features ${f_c}$ and passed through TPABs to refine local texture features and compensate for the loss of high-frequency texture details. Specifically, $\overline{f_o}$ and $\widehat{f_o}$ are processed respectively by residual channel attention branches^8^ and residual local spatial branches^41^ to extract deep local features. Meanwhile, $\overline{f_o}$ and $\widehat{f_o}$ are concatenated with $f_o$ and fused by a convolutional layer. Lastly, the aggregated local features pass through a GTFB to establish connections between local and non-local features.

### Global texture fusion block

Non-local^15,16,42^ operations are capable of capturing long-range dependencies between different parts of an image, addressing the limitation of receptive filed by introducing self-attention mechanisms that enable each position to attend to all other positions in the input data. This operation is particularly instrumental in restoring structural textures that exhibit strong self-similarity. Previous researchers^15,42^ hypothesized that non-local textures with higher similarity scores would be more advantageous for restoring edge information. However, they overlooked an objective fact that when an image suffers from severe degradation, non-local textures with low similarity scores may actually be more useful for restoring edges^16^.

Fusing the local spatial texture features without careful consideration does not significantly improve the network’s ability to restore textures. Therefore, we cascade a global texture feature fusion block (GTFB) at the end of each TPAG. In the module, we adopt the global learnable attention block^16^ after the local feature fusion. The global learnable attention block adaptively adjusts the similarity scores of non-local textures, allowing the network to effectively utilize non-local textures that previously had low similarity scores but can provide rich details.

**Figure 4:**
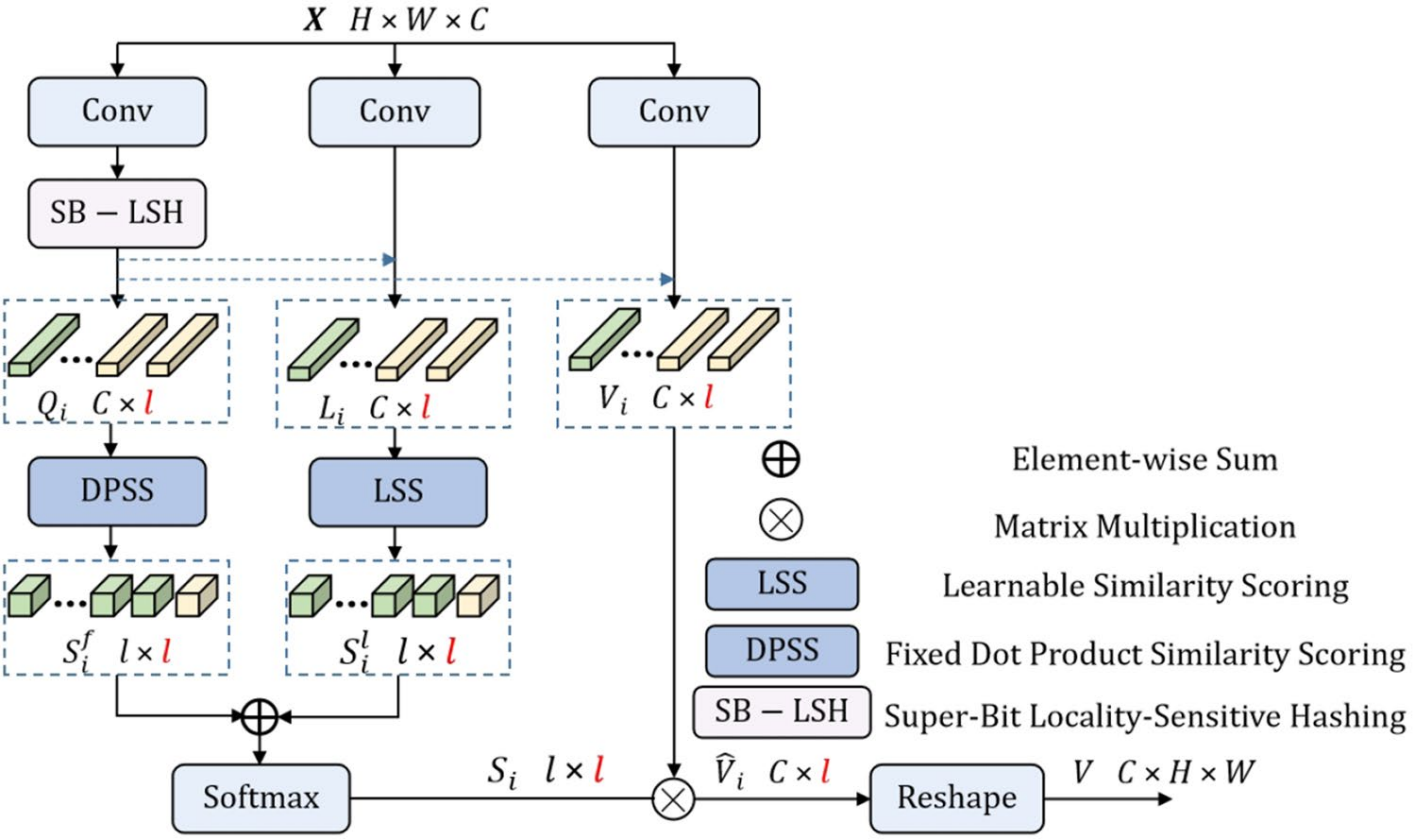
The details about global learnable attention^16^ block.

As shown in Fig. 4, we input the feature map $X\in R^{H\times W \times C}$ as the input and convert *X* into three 1D vectors *Q*, *L* and $V\in R^{C\times HW }$ to achieve global attention mechanism. Super-Bit Locality-Sensitive Hashing (SB-LSH) divides the feature map into buckets to reduce computation costs, as shown in the Eq. (8).

8 $$\begin{aligned} \lambda _i = \left\{ x_j|argmax(MX_i)=argmax(MX_j) \right\} , \end{aligned}$$

where $M \in R^{b\times c}$ is a randomly initialized orthogonal matrix and *b* is the number of hash buckets, $X_i\in R^C$ is the $i-th$ component of $Q_i$, $\lambda _i$ is the index set corresponding to $Q_i$. Next, we use learnable similarity score $X_l$ (LSS) and fixed dot product similarity score $X_f$ (DPSS) to measure self-similarity as Eq. (9).

9 $$\begin{aligned} S(X_i) = S_f(X_i)+S_l(X_i), \end{aligned}$$

where $S_f(X_i)=X^T_i X_i$, $S_l(X_i)$ is defined as Eq. (10).

10 $$\begin{aligned} S_l(X_i) = (W_2\sigma (W_1L[\lambda _i]+b_1)+b_2) , \end{aligned}$$

where $\sigma$ is the ReLU activation and $W_1, W_2, b_1, b_2$ are learnable parameters.

**Table 1:** The quantitative results on benchmarks with Gaussian8 kernels.

Method	Scale	Set5^43^		Set14^44^		BSD100^45^		Urban100^46^		Manga109^47^	
		PSNR	SSIM	PSNR	SSIM	PSNR	SSIM	PSNR	SSIM	PSNR	SSIM
Bicubic	x2	28.82	0.8577	26.02	0.7634	25.92	0.7310	23.14	0.7258	25.60	0.8498
CARN^48^		30.99	0.8779	28.10	0.7879	26.78	0.7286	25.27	0.7630	26.86	0.8606
Bicubic+ZSSR^13^		31.08	0.8786	28.35	0.7933	27.92	0.7632	25.25	0.7618	28.05	0.8769
Deblurring^49^+CARN^48^		24.20	0.7496	21.12	0.6170	22.69	0.6471	18.89	0.5895	21.54	0.7946
CARN^48^+Deblurring^49^		31.27	0.8974	29.03	0.8267	28.72	0.8033	25.62	0.7981	29.58	0.9134
IKC^1^		37.19	0.9526	32.94	0.9024	31.51	0.8790	29.85	0.8928	36.93	0.9667
DANv1^5^		37.34	0.9526	33.08	0.9041	31.76	0.8858	30.60	0.9060	37.23	0.9710
DANv2^2^		37.60	0.9544	33.44	0.9094	32.00	0.8904	31.43	0.9174	38.07	0.9734
DCLS^3^		*37.63*	**0.9554**	*33.46*	**0.9103**	*32.04*	*0.8907*	*31.69*	*0.9202*	*38.31*	*0.9740*
Ours		**37.71**	*0.9548*	**33.56**	*0.9099*	**32.10**	**0.8912**	**31.80**	**0.9214**	**38.81**	**0.9745**
Bicubic	x3	26.21	0.7766	24.01	0.6662	24.25	0.6356	21.39	0.6203	22.98	0.7576
CARN^48^		27.26	0.7855	25.06	0.6676	25.85	0.6566	22.67	0.6323	23.85	0.7620
Bicubic+ZSSR^13^		28.25	0.7989	26.15	0.6942	26.06	0.6633	23.26	0.6534	25.19	0.7914
Deblurring^49^+CARN^48^		19.05	0.5226	17.61	0.4558	20.51	0.5331	16.72	0.5895	18.38	0.6118
CARN^48^+Deblurring^49^		30.31	0.8562	27.57	0.7531	27.14	0.7152	24.45	0.7241	27.67	0.8592
IKC^1^		33.06	0.9146	29.38	0.8233	28.53	0.7899	24.43	0.8302	32.43	0.9316
DANv1^5^		34.04	0.9199	30.09	0.8287	28.94	0.7919	27.65	0.8352	33.16	0.9382
DANv2^2^		34.12	0.9209	30.20	0.8309	29.03	0.7948	27.83	0.8395	33.28	0.9400
DCLS^3^		**34.21**	**0.9218**	*30.29*	*0.8329*	*29.07*	*0.7956*	*28.03*	*0.8444*	*33.54*	*0.9414*
Ours		*34.15*	*0.9213*	**30.40**	**0.8340**	**29.13**	**0.7978**	**28.30**	**0.8491**	**33.92**	**0.9436**
Bicubic	x4	24.57	0.7108	22.79	0.6032	23.29	0.5786	20.35	0.5532	21.50	0.6933
CARN^48^		26.57	0.7420	24.62	0.6226	24.79	0.5963	22.17	0.5865	21.85	0.6834
Bicubic+ZSSR^13^		26.45	0.7279	24.78	0.6268	24.97	0.5989	22.11	0.5805	23.53	0.7240
Deblurring^49^+CARN^48^		18.10	0.4843	16.59	0.3994	18.46	0.4481	15.47	0.3872	16.78	0.5371
CARN^48^+Deblurring^49^		28.69	0.8092	26.40	0.6926	26.10	0.6528	23.46	0.6597	25.84	0.8035
IKC^1^		31.67	0.8829	28.31	0.7643	27.37	0.7192	25.33	0.7504	28.91	0.8782
DANv1^5^		31.89	0.8864	28.42	0.7687	27.51	0.7248	25.86	0.7721	30.50	0.9037
DANv2^2^		32.00	0.8885	28.50	0.7715	27.56	0.7277	25.94	0.7748	30.45	0.9037
AdaTarget^14^		31.58	0.8814	28.14	0.7626	27.43	0.7216	25.72	0.7683	29.97	0.8955
DCLS^3^		**32.12**	*0.8890*	*28.54*	*0.7728*	*27.60*	*0.7285*	*26.15*	*0.7809*	*30.86*	*0.9086*
Ours		*32.07*	**0.8891**	**28.62**	**0.7747**	**27.63**	**0.7304**	**26.31**	**0.7860**	**30.98**	**0.9097**

The best two results are marked in bold and italic, respectively.

**Table 2:** The quantitative comparison on benchmarks with Gaussian8 kernels and various noise levels.

Method	Noise level	Set5^43^		Set14^44^		BSD100^45^		Urban100^46^		Manga109^47^	
		PSNR	SSIM	PSNR	SSIM	PSNR	SSIM	PSNR	SSIM	PSNR	SSIM
Bicubic+ZSSR^13^	15	23.32	0.4868	22.49	0.4256	22.61	0.3949	20.68	0.3966	22.04	0.4952
IKC^1^		26.89	0.7671	25.28	0.6483	24.93	0.6019	22.94	0.6362	25.09	0.7819
DANv1^5^		26.95	0.7711	25.27	0.6490	24.95	0.6033	23.00	0.6407	25.29	0.7879
DANv2^2^		26.97	0.7726	25.29	0.6497	24.95	0.6025	23.03	0.6429	25.32	0.7896
DCLS^3^		*27.14*	*0.7775*	*25.37*	*0.6516*	*24.99*	*0.6043*	*23.13*	*0.6500*	*25.57*	*0.7969*
Ours		**27.29**	**0.7812**	**25.47**	**0.6554**	**25.04**	**0.6075**	**23.45**	**0.6630**	**25.89**	**0.8063**
Bicubic+ZSSR^13^	30	19.77	0.2938	19.36	0.2534	19.43	0.2308	18.32	0.2450	19.25	0.3046
IKC^1^		25.27	0.7154	24.15	0.6100	24.06	0.5674	22.11	0.5969	23.80	0.7438
DANv1^5^		25.32	0.7276	24.15	0.6138	24.04	0.5678	22.08	0.5977	23.82	0.7442
DANv2^2^		25.36	0.7264	24.16	0.6121	24.06	0.5690	22.14	0.6014	23.87	0.7489
DCLS^3^		*25.49*	*0.7323*	*24.23*	*0.6131*	*24.09*	*0.5696*	*22.37*	*0.6119*	*24.21*	*0.7582*
Ours		**25.63**	**0.7369**	**24.32**	**0.6166**	**24.13**	**0.5721**	**22.54**	**0.6222**	**24.24**	**0.7635**

The best two results are marked in bold and italic, respectively.

### Loss function

Our model includes the kernel estimation task and the reconstruction task. We jointly optimize our model using $L_1$ Loss $L_{kernel}$ and Charbonnier Loss $L_{pixel}$, as shown in the Eq. (11).

11 $$\begin{aligned} L_{total} = L_{kernel}+L_{pixel}, \end{aligned}$$

where the $L_{kernel}=||k-k_l||$ is the $L_1$ loss between estimated kernel *k* and the ground truth blur kernel $k_l$. The pixel loss is defined as $L_{pixel}=\sqrt{(I_{SR}-I_{HR})^2+\epsilon }$, where $I_{SR}$ and $I_{HR}$ denote the super-resolved image and the ground-truth HR image, $\epsilon$ is a constant and usually $1\times 10^{-6}$.

## Experiments

### Datasets and implementation details

#### Datasets and metrics

Following previous work^1,2,5^, we used the DIV2K^50^ (800) and the Flickr2K^51^ (2650) as the training data, which together contain 3450 2K HR images. We adopt both isotropic and anisotropic Gaussian kernels as assumed degradation to synthesize corresponding LR images according to Eq. (1). The experimental results are evaluated using the PSNR and SSIM^52^ metrics for fidelity, which are only calculated on the Y channel of the YCbCr color space.

**Figure 5:**
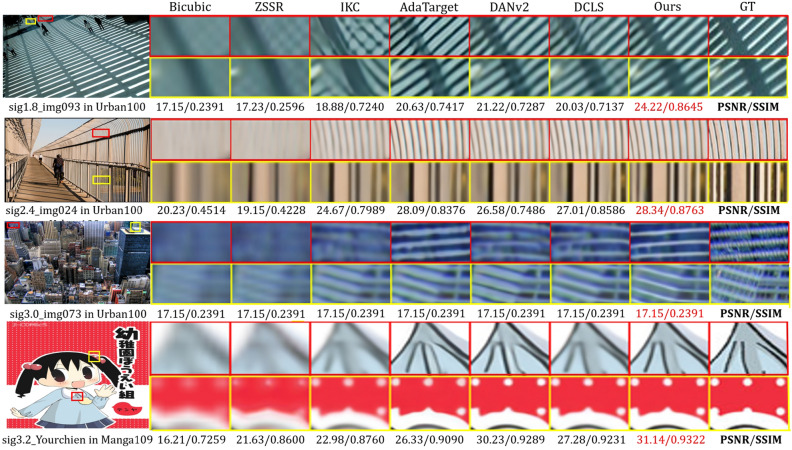
The visual results of sig1.8_img093,sig2.4_img024,sig3.0_img073 in Urban100^46^ and sig3.2_YouchienBoueigumi in Manga109^47^.

#### Isotropic Gaussian kernels

In the setting 1, isotropic Gaussian kernels are first applied in our study as the same in^1–3,5^. The kernel size is fixed to $21\times 21$ during both the training and testing phases. During the training process, we randomly sampled the kernel width from the ranges of [0.2, 2.0] , [0.2, 3.0] , and [0.2, 4.0] uniformly for scale factors of 2, 3, and 4, respectively. During the testing phase, we used Gaussian8 kernels to degrade five benchmarks, including Set5^43^, Set14^44^, B100^45^, Urban100^46^, and Manga109^47^. Gaussian8 uniformly selects 8 kernels from the ranges [0.80, 1.60], [1.35, 2.40], and [1.80, 3.20] for scale factors 2, 3, and 4, respectively. Subsequently, the HR images are convolved with 8 various blur kernels and downsampled to obtain corresponding LR images.

#### Anisotropic Gaussian kernels

In the setting 2, anisotropic Gaussian kernels were employed in our study follwing the work in^1–3,5,9^. The kernel size is $11\times 11$ and $31\times 31$ for scale factors 2 and 4 respectively in the training stages. During the training process, we randomly sampled the kernel width from the ranges of [0.6, 5] and rotated it from the range $[$ $-\pi$ $,$ $\pi$ $]$. During the testing process, blind SR benchmark DIV2KRK^9^ were used for evaluation.

#### Implementation details

We cropped the training data into sub-images of size $480\times 480$, and utilized LR patches of size $64\times 64$ to feed into our model. Our SR network consists of 6 groups of TPAG, each consisting of 11 TPABs and 1 GTFB. We trained the model using 8 RTX2070 GPUs, with a batch size of 4 for each GPU. The initial learning rate was $1\times 10^{-4}$ and decayed by half at every $2\times 10^{5}$ iterations, the total number of iterations was $1\times 10^{6}$. We used the Charbonnier loss^21^ as loss function and Adam^53^ optimizer with $\beta _1$ 0.9 and $\beta _2$ 0.99 for optimization. We also adopt horizontal flipping and $90^{\circ }$ rotation as data augmentation strategies during the training phase.

### Comparison with state-of-the arts

#### Evaluation with isotropic Gaussian kernels

We have evaluated our method on benchmarks synthesized by Gaussian8 kernels and compared its performance with those using state-of-the-art blind SR methods, including ZSSR^13^, IKC^1^, DANv1^5^, DANv2^2^, AdaTarget^14^, KOALAnet^32^, and DCLS^3^. Additionally, CARN^48^ as a lightweight non-blind SR model that combined with blind deblurring^49^ method was also implemented for comparison.

The quantitative comparisons on benchmarks with Gaussian8 kernels are shown in Table 1. Our method achieves remarkable results on various benchmarks, particularly exhibiting noticeable performance on datasets with strong self-similarity, such as Urban100^46^ and Manga109^47^, nearly + 0.16dB and + 0.15dB than DCLS^3^ on $\times$4 factor. Bicubic interpolation and CARN^48^ are non-blind SR methods that assume a known bicubic degradation, which deviates from the actual situation, resulting in a severe drop in performance. ZSSR^13^ utilizes the internal statistics of patch recurrence to build an image-specific super-resolution method that does not require external datasets. This approach slightly improves performance due to the lack of abundant training data and powerful fitting ability. Performing the blind deblurring^49^ operation on the reconstructed image can moderately improve performance by reducing artifacts caused by domain gap. Conversely, applying the inverse operation may further damage details in the LR image, leading to unsatisfactory SR results. The IKC^1^ and DAN^5^ compensate for the offset caused by kernel estimation through iterative correction and end-to-end alternate optimization, respectively, significantly improving the performance. DCLS^3^ can retain the spatial information of the blur kernel while introducing dynamic convolution to boost the robustness of estimation, thus achieving superior performance.

Our proposed TPAB compensates for the attenuation of high-frequency components caused by the DCLS^3^ deconvolution module and the GTFB integrates non-local features with low similarity scores to assist in the fusion of local and global features. The qualitative visual results in Fig. 5 also demonstrate that our method is capable of recovering sharp edges and rich details. Furthermore, considering the complexity of actual degradation, we conduct an extra experiment to handle images with Gaussian8 kernels and additional noise. The quantitative results, shown in Table 2, validate that our method also has a certain degree of robustness to additional noise.

**Table 3:** The quantitative results on DIV2KRK benchmark with isotropic Gaussian kernel.

Method	DIV2KRK^9^			
	x2		x4	
	PSNR	SSIM	PSNR	SSIM
Bicubic	28.73	0.8040	25.33	0.6795
Bicubic+ZSSR^13^	29.10	0.8215	25.61	0.6911
EDSR^7^	29.17	0.8216	25.64	0.6928
RCAN^8^	29.20	0.8223	25.66	0.6936
DBPN^54^	29.13	0.8190	25.58	0.6910
DPBN^54^+Correction^55^	30.38	0.8717	26.79	0.7426
KernelGAN^9^+SRMD^4^	29.57	0.8564	27.51	0.7265
KernelGAN^9^+ZSSR^13^	30.36	0.8669	26.81	0.7316
IKC^1^	–	–	27.70	0.7668
DANv1^5^	32.56	0.8997	27.55	0.7582
DANv2^2^	32.58	0.9048	28.74	0.7893
AdaTarget^14^	-	-	28.42	0.7854
KOALAnet^32^	31.89	0.8858	27.77	0.7637
DCLS^3^	*32.75*	**0.9094**	*28.99*	*0.7946*
Ours	**32.92**	*0.9054*	**29.04**	**0.7982**

The best two results are marked in bold and italic, respectively.

Table 3 shows the quantitative results of these methods on the DIV2KRK^9^ dataset. The results indicates that ZSSR^13^ can serve as a method for improving bicubic interpolation performance. When combined with the kernel estimation by KernelGAN^9^ as a prior, the performance of ZSSR^13^ is further improved. SRMD^4^ shows the consistently with bicubic interpolation. Classical SR methods such as RCAN^8^, EDSR^7^, and DBPN^54^, which adopted paired training data degraded by bicubic downsampling, suffer an extreme decrease in performance due to domain gap. The correction filter^55^ modifies the blurry image to match bicubic kernel, significantly improving the performance of DPBN^54^ trained on bicubic kernel.

Among the remaining blind SR methods, which contain IKC^1^, DAN^2,5^, KOALAnet^32^, AdaTarget^14^,and DCLS^3^, our method performed slightly superior than the DCLS^3^. This circumstance is consistent with our hypothesis. Due to the wild degradation of the DIV2KRK^9^ dataset, the textures and edges are damaged severely. The compensation of TPAB module for high-frequency features is limited. GTFB cannot accurately adjust the similarity score of local textures, resulting in the reconstruction of high-frequency information that is not as good as isotropic Gaussian kernels with mild degradation.

### Ablation study and discussion

**Table 4:** The details of ablation study.

Abalation study	Channel split	Input			Block in each group		
		$f_c$	$\overline{f_o}$	$\widehat{f_o}$	DPAB	TPAB	GTFB
Baseline	$\times$	$\checkmark$	$\checkmark$	$\times$	10	$\times$	$\times$
w.o/ GTFB	$\checkmark$	$\checkmark$	$\checkmark$	$\checkmark$	$\times$	12	$\times$
w.o/ TPAB	$\times$	$\checkmark$	$\checkmark$	$\times$	11	$\times$	1
Ours	$\checkmark$	$\checkmark$	$\checkmark$	$\checkmark$	$\times$	11	1

The SR Network contains five groups that consist of various number of input and blocks based on whether channel split strategy is adopted.

**Table 5:** The ablation study on benchmarks with Gaussian8 kernels.

Baseline	TPAB	GTFB	Params(M)	FLOPs (G)	Inference(s)	Set5^43^		Set14^44^		BSD100^45^		Urban100^46^		Manga109^47^	
						PSNR	SSIM	PSNR	SSIM	PSNR	SSIM	PSNR	SSIM	PSNR	SSIM
$\checkmark$	$\times$	$\times$	13.63	368.15	0.061	**32.12**	*0.8890*	28.54	0.7728	27.60	0.7285	26.15	0.7809	30.86	*0.9086*
$\times$	$\checkmark$	$\times$	21.33	723.72	0.096	32.03	0.8879	*28.56*	*0.7729*	27.60	0.7293	26.15	0.7814	*30.87*	0.9071
$\times$	$\times$	$\checkmark$	15.43	448.77	0.078	31.95	0.8872	28.52	0.7721	*27.61*	*0.7295*	*26.20*	*0.7827*	30.81	0.9074
$\times$	$\checkmark$	$\checkmark$	21.98	747.90	0.108	*32.07*	**0.8891**	**28.62**	**0.7747**	**27.63**	**0.7304**	**26.31**	**0.7860**	**30.98**	**0.9097**

The FlOPs are calculated with input size of 270$\times$180.

In this subsection, we performed a series of ablation experiments on the two crucial modules proposed by us, TPAB and GTFB, to quantitatively study their contributions to our method. The specific settings related to the ablation experiments are shown in the Table 4.

Firstly, the DCLS^3^ adopt clean feature $f_c$ with original $f_o$ as input to feed into Double Path Attention Groups (DPAG) to reconstruct HR images. The DCLS was used as baseline to explore the function of our proposed modules TPAB and GTFB.

Secondly, we placed DPAG with our proposed TPAG, where original feature $f_o$ was split into $\overline{f_o}$ and $\widehat{f_o}$ to extract channel and spatial local feature to compensate for high-frequency decline. In this setting, without the function of global feature fusion, the single GTFB was placed by a TPAB. It can be observed from Table 5 that adding only the TPAB module resulted in a minimal improvement in performance(+ 0.02db in Set14^44^ and + 0.01dB in Manga109^47^). This may be because the depth of TPAG is already sufficient for extracting degradation feature, and using TPAB alone to capture local texture features has limited compensatory effects on high-frequency information.

Lastly, we utilized a variant network consisting of Double Path Attention blocks (DPAB) and Global texture fusion block to evaluate the contribution of GTFB, we appended a GTFB in each DPAG. The results shows a similar trend to the previous experiments, indicating GTFB could better utilize non-local textures to reconstruct high-frequency details. However, due to the lack of tiny compensation from the TPAB module, there is only a moderate performance improvement(about + 0.05dB in Urban100^46^), and the ability to reconstruct texture information was still insufficient.

#### Performance on real degradation

**Figure 6:**
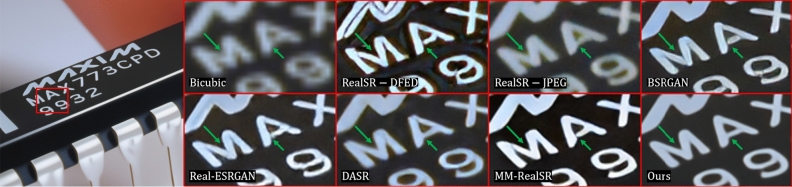
Comparison of real-world image of chip in Real20^11^ dataset for x4 SR. The methods include RealSR^10^, BSRGAN^11^, Real-ESRGAN^12^, DASR^31^, MM-RealSR^56^.

To further demonstrate the effectiveness of our method, we utilized the proposed model with isotropic Gaussian kernels and additional noise level 15 on real degradation images where the degradation is complicated and unknown. Our model was compared with classical real-world super resolution methods including RealSR^10^, BSRGAN^11^, Real-ESRGAN^12^, DASR^31^, and MM-RealSR^56^ on Real20^11^ dataset. An example of super-resolving chip image is shown in Fig. 6. Our method still produce rich details and sharp edges.

## Discussion

The specific results of the ablation experiments are shown in Table 5. It is evident that adding either module alone only results in a marginal performance gain(approximately + 0.05dB in Set14^44^ and BSD100^45^). However, the flexible combination of two modules achieves astonishingly higher performance (+ 0.16dB and + 0.13dB in Urban100^46^ Manga109^47^ respectively than only one module). One possible reason is that even slight compensation of high-frequency information is crucial for the adaptive adjustment of similarity scores in global learnable attention^16^ block. With the aggregation of local features on both channel and spatial dimensions introduced by the TPAG module, the GTFB exhibits a stronger ability to fuse global information.

### Limitation

Our model has achieved good results in super-resolving images with both synthetic degradation and real-world. However, since our training data only covers blurring and noise, without considering more severe and complicated degradation, our model’s performance is not satisfactory when facing images with wild degradation. Meanwhile, due to the dependence on predicting specific kernel parameters, the accuracy of kernel estimation still has a moderate impact on the reconstructed image. We also conducted a comparison of running time and mode size with state-of-the-arts methods, and the results are shown in Table 6. Due to the global information modeling performed by the GLA^16^ module, the computational cost is increased. And channel split strategy increases memory access cost, which is a significant factor affecting inference speed.

## Conclusion

In this work, we propose a blind SR network that is capable of combining kernel estimation with structural prior knowledge. Our method consists of two steps: degradation representation embedding and texture details recovery. A triple path attention block was first proposed to extract local spatial and channel features to compensate for the loss of high-frequency components caused by the first steps.

Subsequently, the global texture fusion block was used to fuse local and global textures, thus providing complementary information for the recovery of HR images. A serious of experiments on benchmarks with different degradation settings demonstrates that our method achieves outstanding performance in blind SR. In future work, we primarily have three main tasks: First, we will utilize contrastive learning to predict the degradation representation of images to disguise different types and levels of degradation, rather than specific parameters of kernel. Second, we will attempt more practical degradation methods to further generalize the model to real-world images.

**Table 6:** The comparison of complexity of different models. The inference latency is tested on RTX3090 GPU.

Method	Params (M)	FLOPs (G)	Inference (s)
IKC^1^	5.29	2178.72	0.503
DANv1^5^	4.33	926.72	0.082
DANv2^2^	4.71	918.12	0.076
DCLS^3^	19.05	368.15	0.061
Ours	27.40	747.91	0.108

The FLOPs are calculated with input size of 270 $\times$ 180.

## Data Availability

The test datasets analyzed during the current study on DIV2KRK and Gaussian8.

## Code availability

The relevant code is made available on this link as open source.
